# Mechanisms of copper homeostasis in bacteria

**DOI:** 10.3389/fcimb.2013.00073

**Published:** 2013-11-05

**Authors:** José M. Argüello, Daniel Raimunda, Teresita Padilla-Benavides

**Affiliations:** ^1^Department of Chemistry and Biochemistry, Worcester Polytechnic InstituteWorcester, MA, USA; ^2^Instituto de Investigación Médica M. y M. Ferreyra, INIMEC-CONICET, Universidad Nacional de CórdobaCórdoba, Argentina

**Keywords:** copper, homeostasis, transmembrane transport, metalloenzymes, metallochaperones, Cu^+^-ATPases

## Abstract

Copper is an important micronutrient required as a redox co-factor in the catalytic centers of enzymes. However, free copper is a potential hazard because of its high chemical reactivity. Consequently, organisms exert a tight control on Cu^+^ transport (entry-exit) and traffic through different compartments, ensuring the homeostasis required for cuproprotein synthesis and prevention of toxic effects. Recent studies based on biochemical, bioinformatics, and metalloproteomics approaches, reveal a highly regulated system of transcriptional regulators, soluble chaperones, membrane transporters, and target cuproproteins distributed in the various bacterial compartments. As a result, new questions have emerged regarding the diversity and apparent redundancies of these components, their irregular presence in different organisms, functional interactions, and resulting system architectures.

## Introduction

**Figure 1:**
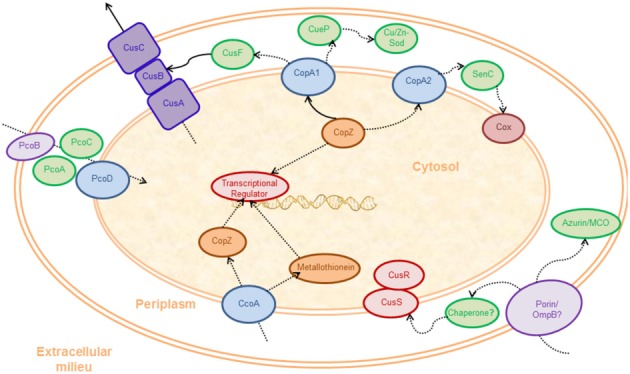
**Scheme of systems participating in Cu^+^ homeostasis in a Gram-negative bacterial cell**. The drawing represents the major systems, and not all cuproenzymes are depicted. Various bacteria contain different subsets of these molecules (Hernández-Montes et al., 2012). Experimentally verified Cu^+^ transfer and transport events are indicated with solid lines. Postulated Cu^+^ fluxes are indicated with dotted lines. The colored shapes represent groups of proteins (various transcriptional regulators, various chaperones, etc.): putative outer membrane transporters (lavender), periplasmic Cu^+^ chaperones and cuproenzymes (green), membrane cuproenzymes (magenta), Cus system (royal blue), inner membrane transporters (purple), transcriptional regulators (red), cytosolic Cu^+^ chaperones (orange).

Copper (Cu) is a micronutrient required as a co-factor in multiple proteins. It participates in redox reactions (electron transport, oxidative respiration, denitrification, etc.) (Silva and Williams, 2001; Cobine et al., 2006; Tavares et al., 2006), and in some cases is also a structural element (Adman, 1991; Kaim and Rall, 1996). Cu homeostatic mechanisms were initially uncovered by phenotypic analysis of bacterial strains carrying mutations in genes participating in Cu tolerance (Odermatt et al., 1993; Outten et al., 2000; Rensing et al., 2000). Perhaps because of the simplicity of experiments measuring Cu tolerance and intracellular Cu accumulation, these mechanisms were the focus of much of the early research in the field. However, cell physiological fitness requires Cu homeostasis mechanisms that primarily address how this metal is distributed and targeted to cuproenzymes. Cells strive to supply Cu to these proteins via compartmentalization involving both, transport across membranes and trafficking within a given compartment (Robinson and Winge, 2010; Argüello et al., 2012). In addition, Cu homeostasis requires chelation by high affinity binding molecules and Cu^+^ sensing by transcriptional regulators to maintain low levels of free Cu, as the metal might participate in a number of deleterious reactions. These include the production of highly reactive radical oxygen species via the Fenton reaction and the interference with [Fe-S] cluster protein assembly (Gaetke and Chow, 2003; Macomber and Imlay, 2009; Dupont et al., 2011). In this review, we focus on these homeostatic mechanisms: sensors, transporters, chaperones, and chelators that distribute the ion to cuproproteins while maintaining beneficial Cu levels (Figure 1).

## Bacterial cuproenzymes

The evolutionary driving force for copper usage in living organisms is mainly represented by the increase of diooxygen on Earth approximately 2 billion years ago (Boal and Rosenzweig, 2009). In parallel, Fe use increased as Fe^2+^/Fe^3+^ equilibrium shifted toward the more oxidized species, leaving the insoluble Fe^3+^ out of the scene. Instead, Cu emerged as a readily available biological redox factor because of the higher solubility of Cu^2+^. Additional determinants for Cu selection over other transition metals are found in its high polarizability and preferential geometries of coordination. Linear and trigonal coordination with S/N-ligands (soft bases), and to a lesser extent with O-ligands (hard base), result in high stability constants of Cu adducts suitable for traffic and transport. On the other hand, static/catalytic Cu sites present higher coordination numbers and more complex geometries with similar stability constants (Boal and Rosenzweig, 2009; Argüello et al., 2012). Most cuproenzymes described in this section harbor one or more Cu sites with tetrahedral or higher geometries. A thorough examination of coordination geometries is beyond the scope of this review.

Only a small repertoire of bacterial cuproenzymes is known (Table 1) (Garcia-Horsman et al., 1994; Claus, 2003; Nakamura and Go, 2005; Claus and Decker, 2006; Ridge et al., 2008; Boal and Rosenzweig, 2009; Rensing and McDevitt, 2013). However, as suggested by bioinformatics studies and in some cases shown by metalloproteomics approaches, bacteria are likely to have many yet unidentified cuproproteins (Ridge et al., 2008; Cvetkovic et al., 2010; Osman et al., 2010; Gladyshev and Zhang, 2013). Early analysis assumed that since most cuproproteins were localized in the bacterial periplasm or plasma membrane (Table 1), there was little need for cytoplasmic cuproproteins metallation. However, consideration of secretion systems might lead to alternative hypothesis. For instance, secretion of cuproenzymes via the Tat system implies that these likely acquire their metal in the cytoplasm. On the other hand, membrane cuproenzymes, as well as soluble enzymes secreted by the Sec system, fold and acquire Cu^+^ from the periplasmic compartment. It can be proposed that this is not a random process. Since considerable energy is spent during protein synthesis, the possibility of wrong metallation must be prevented. That is, highly regulated Cu transport and delivery systems should have co-evolved with the extracytoplasmic cuproenzymes. The participation of cytoplasmic influx and efflux systems in the eventual periplasmic metallation of *cbb_3_* cytochrome *c* oxidase (*cbb3*-COX) provides an example of the necessary passage of Cu^+^ through a controlled delivery system (Figure 1) (González-Guerrero et al., 2010; Ekici et al., 2012b; Lohmeyer et al., 2012).

**Table 1:** Bacterial Cuproenzymes^*^.

**Enzyme**	**Cu^+^ binding sites**	**Cellular localization**	**Secretion mechanism**	**Function**
Cytochrome oxidases				
*cbb3*	1	Inner membrane		Dioxygen reduction
*aa_3_, caa_3_*	2			
Nitrite reductase	2	Inner membrane/periplasmic	Tat^**^	NO^2-^ reduction
Nitric oxide reductase	1	Inner membrane		NO reduction
Nitrous oxide reductase	4	Inner membrane/periplasmic	Tat^**^	N_2_O reduction
Cu,Zn-Superoxide dismutases	1	Secreted/periplasmic/cytoplasmic	Sec^***^	Dismutation of O^−^_2_
Plastocyanin	1	Thylakoid	Sec^***^	Electron transfer
Azurin	1	Periplasmic	Sec^**^	Electron transfer
Laccases	4	Periplasmic	Tat^**^	Phenols and diamines oxidation
CueO	2-4	Periplasmic	Tat^***^	Substrate oxidation
NADH dehydrogenase-2	1	Inner membrane		NADH oxidation
Tyrosinases	1	Secreted	Tat^***^	Monophenol hydroxylation
Particulate methane monoxygenases	2	Membrane		Methane oxidation
Amine oxidases	1	Periplasmic/secreted	Tat^**^	Oxidative deamination
Polysaccharide oxygenases	1	Secreted	Tat^***^	Cellulose oxidation

* *References are in the text*.

** *Based on the presence of signal sequences*.

*** *Based on experimental evidence*.

Heme-Cu respiratory oxidases constitute a large superfamily of cuproenzymes present in most bacteria (Garcia-Horsman et al., 1994; Richter and Ludwig, 2003). They are responsible for the reduction of O_2_ and the generation of the H^+^ electrochemical gradient. The O_2_ reduction takes place in the binuclear center of subunit I (also referred as subunit N). This reduction center is composed of a heme iron juxtaposed to a Cu site (Cu_B_). In addition, some *ba_3_*, *caa_3_* and *aa_3_* oxidases, contain a Cu_A_ center in subunit II (Garcia-Horsman et al., 1994). Terminal oxidases acquire their Cu cofactors from periplasmic Cu^+^-chaperones. Homologs of the Cu^+^-chaperones ScoI/SenC have been proposed to perform this function in *Pseudomonas putida*, *Rubrivibax gelatinosus*, and *Rhodobacter capsulatus* as the corresponding deletion mutants lack *cbb3*-COX activity (Banci et al., 2011; Ekici et al., 2012a; Lohmeyer et al., 2012). Studies in *Pseudomonas aeruginosa* revealed the requirement of cytosolic Cu^+^ efflux through CopA2-like ATPases for Cu^+^ insertion into *cbb3*-COX (González-Guerrero et al., 2010). Thus, it is likely that ScoI/SenC chaperones receive the metal from the ATPase. The identification of *R. capsulatus* CcoA has further supported the idea that cytoplasmic Cu^+^ is channeled to the periplasm for COX metallation (Ekici et al., 2012b). CcoA is a member of the major facilitator superfamily (MFS) of transport proteins. It has been proposed that it imports Cu into the cytoplasm and is required for *cbb_3_* assembly. Regarding oxidases containing a Cu_A_ site, characterization of the *Bradyrhizobium japonicum aa_3_* oxidase suggests that the metal binding PcuC acts as the periplasmic Cu^+^-chaperone (Serventi et al., 2012). The source of Cu for PcuC loading has not been identified.

Three enzymes involved in the bacterial denitrification pathway (i.e., the nitrite reduction to dinitrogen) are periplasmic cuproenzymes: nitrite reductase (Nir), nitric oxide reductase (qCuNOR), and nitrous oxide reductase (N_2_OR) (Messerschmidt et al., 1993; Brown et al., 2000; Zumft, 2005). Nir and N_2_OR are attached to the inner membrane. Nir is homotrimeric with each subunit containing type 1 and 2 Cu centers. N_2_OR is homodimeric with a binuclear Cu_A_ site related to electron transfer and a multinuclear Cu_Z_ related to catalysis (Brown et al., 2000). It is assumed that these three enzymes are exported via the Tat secretion system (Saunders et al., 2000; Berks et al., 2003). However, Nir is apparently secreted via Tat in some organisms and via Sec in others (Berks et al., 2000b). In any case, it has been proposed that Nir proteins present one Cu^+^ binding site at the subunits interface with metal ligands located in two separate polypeptides. Cu would bind these sites during or after oligomer formation in the periplasm (Godden et al., 1991; Berks et al., 2003). Similarly, it has been reported that assembly of the Cu_A_ and Cu_Z_ center of the *Pseudomonas stutzeri* N_2_OR occurs in the periplasm and *nos* genes are required for metallation (Wunsch et al., 2003).

Members of the superoxide dismutase (Sod) harbor different active redox metallic co-factors in their catalytic centers. Two major groups have been described in bacteria, SodA containing usually Mn^2+^ (and in some cases Fe^2+^), and SodC carrying Cu^+^ and Zn^2+^. Most bacterial Cu,Zn-SodC are periplasmic proteins. These are secreted unfolded by the Sec secretion system and likely acquire the metals in the periplasm (Kroll et al., 1995; Imlay and Imlay, 1996). Recent studies of *Salmonella enterica sv*. Typhimirium Cu,Zn-Sod have identified some of the proteins involved in periplasmic metallation. *Salmonella* virulent strains contain SodCI and SodCII (Fang et al., 1999; Krishnakumar et al., 2007; Rushing and Slauch, 2011). These are both periplasmic proteins. SodCI appears required for virulence. *Salmonella* mutant strains lacking functional Cu^+^-ATPases, CopA, and GolT, have inactive SodCII (Osman et al., 2013). Mutation of the periplasmic Cu^+^-chaperone CueP also results in inactive SodCII forms. Consequently, it was hypothesized that Cu^+^ might be transferred from either CopA or GolT to CueP to be inserted in SodCII. Further studies are necessary to understand the apparent redundancy of both ATPases, as well as the Cu transfer mechanism among the involved proteins. Conversely, in *Mycobacterium tuberculosis* and *Mycobacerium smegmatis*, the Cu,Zn-Sod is located in the cytosol whereas the Fe/Mn-SodA is secreted via the SecA2 pathway and is metallated outside the cell (Braunstein et al., 2003; Padilla-Benavides et al., 2013a). Therefore, these organisms require a cytoplasmic Cu-loading mechanism to produce functional Cu,Zn-Sod.

The superfamily of cupredoxins is characterized by an antiparallel β-barrel structure that presents a type 1 Cu binding site. This group includes plastocyanin located in the thylakoid, and a variety of periplasmic proteins such as azurin, multicopper oxidases (MCO), laccases, and nitrosocyanin (Redinbo et al., 1994; Donaire et al., 2002; Berks et al., 2003; Zaballa et al., 2012). In cyanobacteria, plastocyanin participates in the electron shuttling to photosystem I (Redinbo et al., 1994) and also confers protection against Cu^+^ stress (Tottey et al., 2012). Mutant strains lacking Cu^+^-ATPases PacS or CtaA have impaired photosynthetic electron transport via plastocyanin and cytochrome oxidase activity, suggesting that these transporters are required for the metallation of these proteins (Tottey et al., 2001). Periplasmic azurins participate in electron shuttling for deamination and denitrification processes by donating electrons to nitrite reductases (De Rienzo et al., 2000). *P. aeruginosa* azurin participates in the cellular response to Cu stress. For instance, mutation of *P. aeruginosa cinA*, an azurin/plastocyanin-like protein, leads to increased sensitivity to Cu^2+^ in the media (Elguindi et al., 2009). Deletion of either *P. aeruginosa* Cu^+^-ATPase, CopA1, or CopA2, induces an increase in azurin transcription as a cellular response to Cu^+^-derived oxidative stress (Raimunda et al., 2013). However, whether azurins are metallated by the transporters or participate in Cu^+^ oxidation has not been established.

Ascorbate oxidases, laccases, and ceruloplasmin are homologous small blue MCO (Messerschmidt et al., 1993; Nakamura et al., 2003). Laccases, for instance, are involved in the oxidation of phenolic compounds (Claus, 2003). Laccases and ascorbate oxidases present a mononuclear type 1 Cu site -homologous to other cupredoxins- and a trinuclear Cu center. Ceruloplasmin has three mononuclear Cu sites and a trinuclear Cu domain (Nakamura et al., 2003). CueO and PcoA are MCO up-regulated by high Cu^+^ concentrations through the *cueR* regulon (Outten et al., 2000). It has been observed that *Escherichia coli* CueO is folded in the cytoplasm and exported via the Tat system (Outten et al., 2000; Grass and Rensing, 2001). Based on differences in Cu^+^ amounts relative to the binding capacity of CueO, it was proposed that they contribute to periplasmic metal tolerance by oxidizing the ion to the less toxic form Cu^2+^ (Roberts et al., 2002; Singh et al., 2004). However, *S. enterica sv*. Typhimurium CueO seems to also play a significant role in Cu^+^ homeostasis under anaerobiosis (Espariz et al., 2007).

NADH dehydrogenase-2 (NDH-2), part of the electron transport chain, is a membrane bound protein that catalyzes the electron transfer from NADH to quinone (Jaworowski et al., 1981; Bjorklof et al., 2000). NDH-2 Cu^2+^-reductase activity was described in *E. coli* (Rodríguez-Montelongo et al., 1995; Rapisarda et al., 1999). Although not essential for Cu metabolism, NDH-2 is likely important for cell growth under Cu stress (Rodríguez-Montelongo et al., 2006). Moreover, *E. coli* NDH-2 presents four domains. Domain IV likely anchors the protein to the membrane. Domain III faces the cytosolic side near the FAD-binding site, shares homology with the cytosolic metal binding domains (MBD) of Cu^+^-ATPases and might be relevant for cytoplasmic Cu^+^ sensing (Rapisarda et al., 2002). The biological role for the Cu^+^-reducing enzymatic activity, as well as the characterization of the secretion mechanism and metallation of this enzyme, requires further investigation.

In eukaryotic cells, tyrosinase is required for synthesizing melanin. Some bacteria from the genus *Streptomyces* produce a melanin-like pigment (Katz et al., 1983; Hintermann et al., 1985; Huber et al., 1985; Ikeda et al., 1996). Tyrosinases catalyze the orthohydroxylation of monophenol and the subsequent oxidation of the diphenolic product to the resulting quinone. The quinone product is a reactive precursor for the synthesis of melanin pigments. Tyrosinases contain a flexible dinuclear Cu center required for catalysis (Matoba et al., 2006). Bacterial tyrosinase is encoded in a bicistronic operon composed by genes coding for a “caddie” chaperone protein (*melC1*) and tyrosinase (*melC2*) (Katz et al., 1983; Ikeda et al., 1996). Tyrosinases are secreted via the Tat pathway (Berks et al., 2003). MelC1 appears to function as a Cu^+^-chaperone, forming a transient complex with the apo-MelC2. This would facilitate the incorporation of Cu^+^ and the subsequent secretion of functional tyrosinase (Chen et al., 1992, 1993; Matoba et al., 2006).

Methane monooxygenases (MMO) are Cu^+^-containing enzymes present in methanotropic bacteria. There are two MMO forms, membrane-bound (pMMO) and cytosolic (sMMO). Most organisms present the pMMO form; however, in some bacteria both types are present. Interestingly, the expression of both proteins is regulated by the availability of Cu (Nielsen et al., 1997). The *Methylococcus capsulatus* (Bath) pMMO comprises three subunits, encoded by the *pmoB*, *pmoA*, and *pmoC* genes (Semrau et al., 1995; Stolyar et al., 1999). Copper stoichiometries ranging from 2 to 15 ions per αβγ complex have been reported (Nguyen et al., 1998; Basu et al., 2003). Ions appear organized in multiple trinuclear clusters composed of one Cu^2+^ and two Cu^+^ ions (Nguyen et al., 1996, 1998). These sites are hypothesized to function for catalysis and electron transfer. Interestingly, the uptake of methanobactin-bound Cu via TonB dependent transport appears critical for pMMO metallation (Balasubramanian and Rosenzweig, 2008).

Cu^+^-dependent amine oxidases (CuAO) are rare in bacteria but they provide the ability to obtain carbon and nitrogen from primary amines by oxidative deamination (Wilmot et al., 1997). The enzyme is induced in conditions where biogenic primary amine substrate is the sole source of carbon (Gladyshev and Zhang, 2013). Bacterial Cu^+^-dependent polysaccharide oxygenases (AA10) are secreted proteins involved in breaking internal linkages in plant cellulose (Levasseur et al., 2013). AA10 binds one Cu^+^ atom with high affinity. The metal ion is coordinated in a T-shaped configuration by three N atoms from two His side chains and the amino terminus (Hemsworth et al., 2013). Although these enzymes are secreted via the Tat pathway (Berks et al., 2003), there is no information regarding their metallation mechanisms.

This assessment of various cuproenzymes makes clear that they fulfill various roles essential for bacterial survival. It is also apparent that well-regulated mechanisms should be responsible for delivering the metal to final targets. Relevant questions immediately emerge when considering Cu delivery models. Are chaperones promiscuous in their interactions with various targets? If they are specific, how is the allocation of Cu to the various targets regulated? Moreover, the existence of novel uncharacterized cuproproteins must be also considered. Recent efforts using metalloproteomic approaches support this idea. For instance, liquid chromatography, high-throughput tandem mass spectrometry (HT-MS/MS) and inductively coupled plasma mass spectrometry (ICP-MS) were combined to identify cytoplasmic metalloproteins in the extremophile *Pyrococcus furiosus* (Cvetkovic et al., 2010). Chromatography fractions revealed 343 metal peaks. Among these, 158 did not correspond to any predicted metalloprotein, supporting the likelihood of numerous novel cuproproteins.

## Maintaining the Cu^+^ quota: Cu^+^-sensing and transcriptional regulation of homeostatic systems

Cells control alkali ion levels regulating transmembrane transport through various mechanisms: (a) modulating transport turnover rates through chemical modification and allosteric ligand binding; (b) managing the incorporation and removal of transporter proteins at the required membrane (eukaryotes); and (c) transcriptional regulation of transporter abundance. The high affinity binding of transition metals to their transporters, as well as the inability of apo-chaperones to accept Cu^+^ back from the exporting ATPase, makes the transmembrane transport functionally irreversible. Consequently, bacterial cells regulate this process mainly through transcriptional control by modifying the transporter abundance. This strategy facilitates the co-regulation of chaperone and chelating metallothioneins. Four families of Cu^+^-sensing homodimeric transcriptional regulators have been identified. MerR is a transcriptional activator, while CsoR, CopY, and ArsR are transcriptional repressors (Ma et al., 2009c).

CueR-like proteins, members of the MerR family, are present in most proteobacteria (Ma et al., 2009c). These activate the transcription of Cu^+^-ATPases (CopA) and Cu^+^ chaperones (CopZ, CueP, etc.) in response to high concentrations of Cu^+^ (Outten et al., 2000; Grass and Rensing, 2001; Pontel and Soncini, 2009). In the presence of low metal levels, the metalloregulator binds to DNA in a conformation that prevents the DNA-RNA polymerase interaction, therefore repressing transcription (Ma et al., 2009c; Reyes-Caballero et al., 2011). When cytosolic Cu^+^ level increases, metal binding to the sensor induces changes in the DNA binding region. The long-range communication between metal and DNA binding sites appears mediated by a hydrogen bond network. Once the cytosolic Cu^+^ levels return to normal, the metal-resistance genes are down regulated to basal levels. It is possible that the metal is released from the sensor-DNA complex followed by disassociation of the regulatory complex. However, this is an unlikely phenomenon considering the high Cu^+^ binding affinity of CueR (10^−21^ M) (Changela et al., 2003). Rather, the exchange of apo- and holo-forms appears possible since both forms of MerR proteins interact with DNA with similar affinities (Brown et al., 2003; Joshi et al., 2012). Although CueR and other Cu^+^ sensing regulators bind the metal with high affinity, metal selectivity appears to be conferred by the singular coordination geometry of binding sites and the capability to induce the required allosteric changes to influence DNA conformations (Ma et al., 2009c; Reyes-Caballero et al., 2011). CueR-like sensors bind Cu^+^ with two Cys in two symmetrical loops in the periphery of the dimer. Nevertheless, the detail structure of the loop and the influence of the second coordination sphere are critical as shown by selectivity changes associated with minimal modifications in the region (Checa et al., 2007).

CsoR was identified as the Cu^+^ sensor in *M. tuberculosis* (Liu et al., 2007). This Cu^+^-responsive repressor controls the expression of the *cso* operon (*csoR, Rv0968*, the Cu^+^-ATPase *ctpV*, and *Rv0970*). Members of this family are widely distributed in most bacterial species (Smaldone and Helmann, 2007; Ma et al., 2009a; Sakamoto et al., 2010; Corbett et al., 2011). In CsoR, Cu^+^ is bound in a trigonal coordination by two Cys and one His (Liu et al., 2007; Ma et al., 2009b). These sites might also bind Ni^2+^, Zn^2+^, or Co^2+^, but these place the sensors in non-active conformations (Ma et al., 2009a). Thus, as in the case of CueR–like proteins, binding geometry appears critical for selectivity.

Members of the CopY family are present in Firmicutes. *Enterococcus hirae* CopY regulates the *copYZBA* operon, where *copZ* encodes a Cu^+^-chaperone, *copA* a CopA1-like ATPases (see below) and *copB* is a Cu^2+^-ATPase (Argüello et al., 2007; Solioz et al., 2010). Both ATPases mediate the efflux of cytoplasmic Cu^+/2+^ (Raimunda et al., 2011). A conserved CXCXXXCXC motif appears to mediate the binding of two Cu^+^ per CopY monomer. CopY is also interesting because its capability to exchange Cu^+^ with the cytoplasmic Cu^+^-chaperone CopZ has been demonstrated (Cobine et al., 1999). This has not been shown for other Cu^+^ sensors.

*Oscillatoria brevis* BxmR is the only identified Cu^+^ sensor member of the ArsR family (Liu et al., 2004, 2008). This binds Ag^+^ and Cu^+^ through formation of a binuclear Cu_2_S_4_ cluster similar to that of *E. hirae* CopY. Like other described sensors, it regulates the expression of a metallothionein and a Cu^+^-ATPase.

Less is known about the role of bacterial two-component systems in the regulation of Cu^+^ homeostatic systems. The two-component system CusRS regulates the *cusCFBA* system under anaerobic conditions (Outten et al., 2001; Rensing and Grass, 2003; Yamamoto and Ishihama, 2005; Gudipaty et al., 2012). This is a three-component channel/pore that controls periplasmic Cu^+^ (Outten et al., 2001; Rensing and Grass, 2003). The Cus complex is composed by a plasma membrane energy-providing channel, CusA; an outer membrane pore, CusC; CusB, a periplasmic protein linking CusA and CusC; and a soluble periplasmic Cu^+^-chaperone, CusF (Outten et al., 2001; Rensing and Grass, 2003). In the case of CusRS, the periplasmic sensor domain of the transmembrane histidine kinase CusS binds Cu^+^. This drives the subsequent activation of the cytoplasm regulator CusR. The *pcoABCDE* cluster also appears to be regulated by a two-component system, PcoRS (Rouch and Brown, 1997; Munson et al., 2000). The Pco proteins, whose function is not fully understood, appear to contribute to the control of periplasmic Cu^+^ in *E. coli* and other Gram-negative bacteria (Brown et al., 1995; Rouch and Brown, 1997; Lee et al., 2002; Rensing and Grass, 2003; Djoko et al., 2008; Hernández-Montes et al., 2012). Sequence analysis and experimental evidence suggest that PcoA is a periplasmic MCO. PcoB may function as the outer membrane transporter, while PcoD appears to be the inner membrane transporter that drives Cu^+^ entry from the periplasm to the cytoplasm. PcoC is a periplasmic Cu^+^-chaperone, and PcoE is an additional putative chaperone.

## Cu^+^ transport systems

### Cu^+^-transporting P-type ATPases

**Figure 2:**
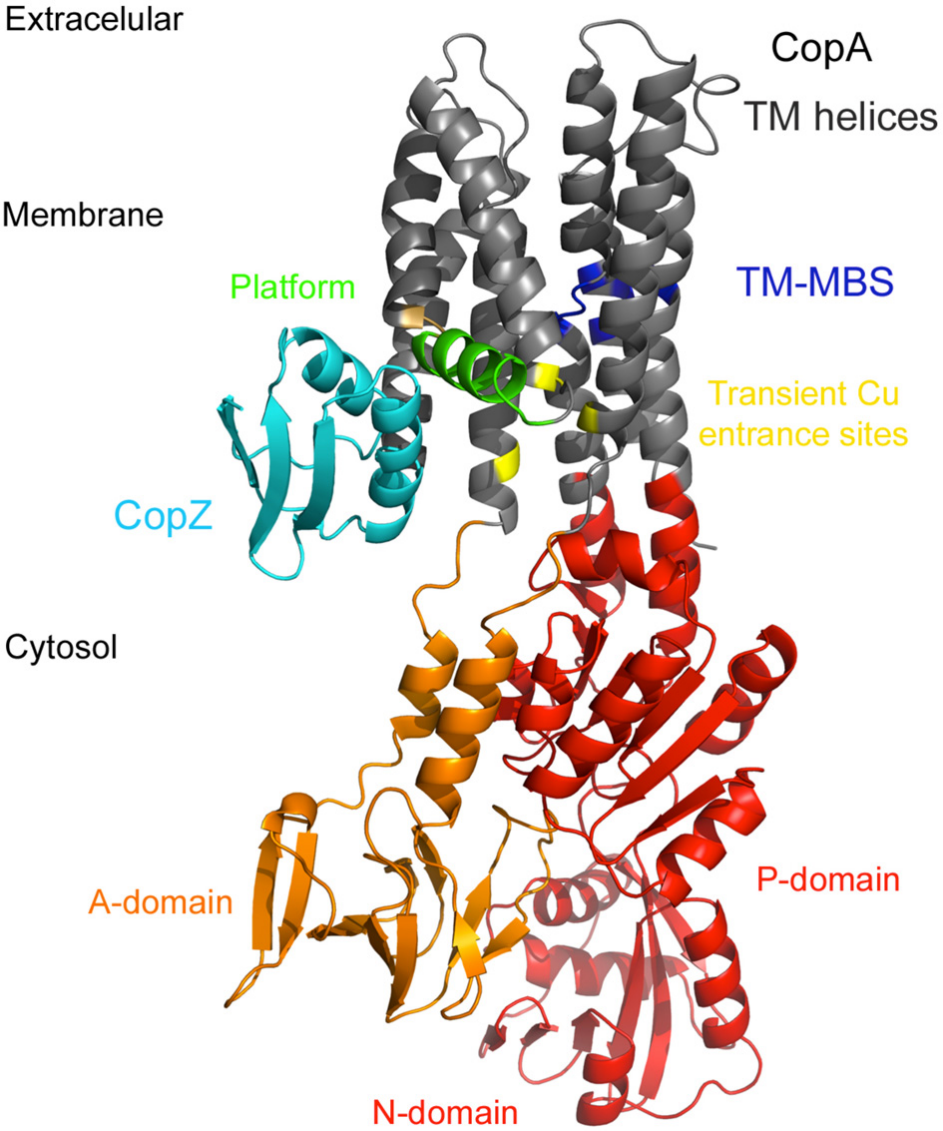
**Structures and interaction between cytosolic Cu^+^-chaperones and Cu^+^-ATPases**. *Archeoglobus fulgidus* C-terminal Cu^+^ binding domain of CopZ modeled using *Enterococcus hirae* CopZ (Protein Data Bank code 1CPZ) in light *blue*. *A. fulgidus* CopA modeled using *Legionella pneumophila* CopA (Protein Data Bank code 3RFU) as a template. The transmembrane segments (TM) are in *gray*, the electropositive platform helix is highlighted in *green*, the Cu^+^ entrance site aminoacids are in *yellow*, the transmembrane metal binding sites (TM-MBS) in *dark blue*, the actuator (A) domain in *orange*, the nucleotide (N) binding and (P) phosphorylation domain in *red*.

The prevailing Cu^+^ transmembrane transporters throughout the bacterial kingdom are P_IB1_-type ATPases (Ridge et al., 2008; Hernández-Montes et al., 2012). These are polytopic membrane transporters that couple the unidirectional Cu^+^ efflux to the hydrolysis of ATP. Initial functional characterization of Cu^+^-ATPases showed that they are responsible for maintaining cytosolic Cu^+^ levels (Rensing et al., 2000; Argüello et al., 2007; Osman and Cavet, 2008; Solioz et al., 2010). The mechanism coupling the ATP hydrolysis to Cu^+^ translocation appears to follow the classical features of the Post-Albers catalytic cycle. This describes solute transport by well-characterized P-type ATPases (Palmgren and Nissen, 2011; Raimunda et al., 2011). Perhaps the most significant differences from alkali metal transport ATPases are those related to the substrate access to transport sites (Figure 2). That is, free Cu^+^ is absent in the cytoplasm and reaches the transmembrane sites after delivery by Cu^+^-chaperones. This substrate transfer is mediated by ligand exchange following protein-protein interactions (González-Guerrero and Argüello, 2008; González-Guerrero et al., 2009; Raimunda et al., 2011). The ATPase transmembrane metal binding sites (TM-MBS) bind two Cu^+^ with extremely high affinities (González-Guerrero et al., 2008). This high affinity, together with inability of the apo-chaperone to remove Cu^+^ from the TM-MBS prevents the backward release of Cu^+^ into the cytoplasm. It is hypothesized that the enzyme releases Cu^+^ to the extra-cytoplasmic compartments in a similar fashion; i.e., delivering the metal to an appropriate chaperone (Raimunda et al., 2011). Additional unanswered aspects of transport is whether another ion, like H^+^, is counter transported and whether Cu^+^ transport is electrogenic. However, considering the general low Cu^+^ transport rate of Cu^+^-ATPases the exchanged mass of the counter ion might not be significant enough to alter any metabolic process.

The crystal structure of *Legionella pneumophila* CopA shows the arrangement of the distinct elements of Cu^+^-ATPases (Gourdon et al., 2011) (Figure 2). The cytoplasmic ATP-binding domain (ATP-BD) is comprised of the phosphorylation (P-), nucleotide binding (N-), and actuator (A-) domains. Within the P-domain is the conserved DKTGT sequence. Phosphorylation of the aspartyl residue during the enzyme catalytic cycle is the hallmark of the P-type ATPase family of transporters. Importantly, six invariant residues in the TM region constitute the TM-MBS and are the determinants of Cu^+^ specificity (Argüello, 2003; González-Guerrero et al., 2008). Besides bacterial Cu^+^-ATPases contain one or two cytosolic N-terminal metal binding domains (N-MBD) (Argüello, 2003). N-MBDs are structurally similar to the Atx1-like family of Cu^+^-chaperones (Boal and Rosenzweig, 2009). These domains exchange Cu^+^ with cytoplasmic chaperones with K_eq_ ≈ 1 (Argüello et al., 2007; Boal and Rosenzweig, 2009; Banci et al., 2010a,b; Robinson and Winge, 2010). Therefore, they sense the levels of cytoplasmic Cu^+^ and regulate the ATPase turnover rate through a Cu^+^-dependent interaction with the catalytic soluble domains (Mandal and Argüello, 2003; González-Guerrero et al., 2009). The structure of *L. pneumophila* CopA also showed a unique feature on the cytosolic side of the second TM helix: a Gly-Gly kink that exposes an electropositive platform to the cytoplasm, as well as three conserved residues that constitute the Cu^+^ “entrance” site (Met, Glu, Asp) (Gourdon et al., 2011). The platform is where Cu^+^-loaded chaperones interact with the transporter while delivering the ion for transport (Padilla-Benavides et al., 2013b). These conserved “entrance” residues are required to take Cu^+^ from the chaperone via ligand exchange. Cu^+^ ions only transiently interacts with these residues during transfer to the TM-MBS, where they reside until ATP hydrolysis drives the necessary E1PE2 transition associated with ion translocation.

### Cus system

The Cus system, first identified in *E. coli*, is proposed to transport cytosolic Cu^+^ across the cell membrane toward the extracellular milieu (Figure 1). This efflux system is comprised of a proton-substrate carrier (CusA) and an outer membrane pore (CusC), which are joined in the periplasm by a linker protein, CusB (Zgurskaya and Nikaido, 1999; Su et al., 2009; Kim et al., 2011). A forth component, CusF is a periplasmic Cu^+^-chaperone found in some organisms (Kim et al., 2011; Mealman et al., 2011; Hernández-Montes et al., 2012).

Comparative studies of CopA and Cus proteins expression have suggested that Cus is not the principal Cu^+^ efflux system in cells growing under aerobiosis (Franke et al., 2001; Outten et al., 2001). This model considers that CopA's role is in fact supported by the periplasmic oxidase CueO. Thus, under anaerobic conditions where CueO would be inactive, Cus would become relevant in Cu^+^ detoxification (Outten et al., 2001). In agreement with this idea, bioinformatics analyses showed that the Cus system coexists with CopA in 44% of the organisms of the γ-proteobacteria group (Hernández-Montes et al., 2012). Among these, the pair CusA/CusC seems to be essential for the assembly of the efflux system, since CusB is absent in most members of this class. Then, CusB may be a dispensable accessory protein for the formation of the CusA-CusC channel, or homologous proteins from other RND complexes may fulfill its function (Hernández-Montes et al., 2012). The periplasmic chaperone CusF is the least conserved element of the Cus system (Hernández-Montes et al., 2012). CusF is a small (10 kDa) protein which binds one Cu^+^ atom in a tetragonal coordination with two Met, one His, and a conserved Trp. The Trp residue stabilizes the Cu^+^ binding, thus regulating metal transference and preventing redox reactions (Xue et al., 2008). Yeast two-hybrid assays and NMR analysis have shown that CusF interacts with both CusB and CusC (Franke et al., 2003; Mealman et al., 2011). Consequently, a proposed transport mechanism is that CusF delivers periplasmic Cu^+^ to CusABC for extracellular transport (Kim et al., 2011; Mealman et al., 2011). However, the transport of cytoplasmic Cu^+^ through the CusA antiporter has also been postulated based upon the CusA structure (Fu et al., 2013). Transport experiments are clearly necessary to discriminate among these alternative hypothetical Cus transport mechanisms.

### Pco system

Pco systems were the first characterized genetic determinants of bacterial Cu resistance (Tetaz and Luke, 1983; Bender and Cooksey, 1986; Cha and Cooksey, 1991). An *E. coli* strain showing a 7-fold increase in Cu resistance was isolated from a piggery that fed its animals a high Cu diet to promote growth. The phenotype was associated with the plasmid pRJ1004. This enabled the *E. coli* transconjugants to thrive in media containing up to 20 mM Cu^2+^ (Tetaz and Luke, 1983). Similarly, increased Cu resistance in *Pseudomonas syringae* pv. *tomato* strains was shown to be determined by the plasmid pPT23D (Cooksey, 1987, 1990). Both plasmids contain 6–7 clustered genes, *pcoABCDRSE* in *E. coli* and *copABCDRS* in *P. syringae* (Mellano and Cooksey, 1988b; Brown et al., 1995). PcoB/CopB and PcoD/CopD are membrane proteins located in the outer and inner membranes respectively (Figure 1), participating in Cu transport. PcoA/CopA and PcoC/CopC are soluble periplasmic Cu^+^-binding proteins (Cha and Cooksey, 1991, 1993; Lee et al., 2002). Initial characterization in *P. syringae* pv. *tomato* pointed that *copA* and *copB* are necessary to confer partial Cu^+^ resistance, whereas *copC* and *copD* genes are required for full resistance (Mellano and Cooksey, 1988a). However, indirect evidence provided by phenotypic characterization of *copC* and *copD* mutant strains suggests that these genes are required for Cu-uptake across the cell membrane. Cells expressing both proteins were hypersensitive toward Cu, and the presence of any of these two genes leads to slight Cu accumulation (Cha and Cooksey, 1993). Direct metal transport determinations are necessary to establish the role of these proteins in Cu homeostasis. The PcoA sequence shows homology to MCO, binds Cu, and has MCO activity (Djoko et al., 2008) and is predicted to be translocated into the periplasm via the Tat pathway (Berks et al., 2000a; Lee et al., 2002). The PcoC structure shows 2 solvent-exposed Cu binding sites and its participation in Cu handling and transferring in the periplasm has been suggested, although its interacting client proteins remain unknown (Arnesano et al., 2002b, 2003). *PcoE* is homologous to the *Salmonella* silver resistance gene *silE* (Cha and Cooksey, 1993; Gupta et al., 2001). It has been postulated that PcoE might provide initial sequestration of Cu in the periplasm before the remaining genes of the *pco* system are fully induced (Lee et al., 2002). *Bacillus subtilis ycnJ* encodes a protein with high homology to domains found in PcoC and PcoD (Chillappagari et al., 2009). The presence of the Cu^+^-chelator BCA in the media led to reduced cytoplasmic Cu, induction of *ycnJ* expression, and impaired growth of an *ycnJ* deletion mutant strain, suggesting a role for YcnJ in Cu import. Importantly, amino acids involved in the Cu^2+^ binding of CopC are conserved in YcnJ sequence (Arnesano et al., 2003; Chillappagari et al., 2009).

Although counterintuitive when considering a Cu resistance mechanism, the biological function of Pco systems seems to be related to periplasmic, and probably, intracellular Cu storage pools (Figure 1) (Cooksey, 1993). The latter function is probably mediated by CopC/CopD pair. However, the *pco* system is not able to rescue the Cu sensitive phenotype in the *copA* mutant of *E. coli*, suggesting that cooperation between CopC/CopD-dependent Cu pools and CopA1-like ATPases is necessary to attain high Cu resistance (Lee et al., 2002).

### Putative outer membrane channel: MctB

MtcB (*Rv1698*) was first characterized as a high conductance channel located in the complex outer membrane of *M. tuberculosis* (Siroy et al., 2008). Expression of MctB in *M. smegmatis* resulted in an increased uptake of carbon source nutrients. While MctB structure is unknown, a functional analogy with Gram-negative porins has been suggested (Siroy et al., 2008). Analysis of the *M. tuberculosis* deletion mutant Δ*mctB* showed decreased Cu^+^ resistance and intracellular Cu^+^ accumulation (Wolschendorf et al., 2011; Rowland and Niederweis, 2012). Importantly, infection experiments in mice and guinea pigs support the hypothesis that MctB is required for *M. tuberculosis* to attain maximal virulence (Wolschendorf et al., 2011). This is likely due to MctB's ability to counteract the bactericidal effect produced by the Cu^+^ overload in activated macrophage phagosomes. Supplementary structural and biochemical studies might define the role of MctB in *M. tuberculosis* Cu^+^ homeostasis and its interrelation with other Cu^+^ efflux systems.

### Cytoplasmic Cu^+^ influx

Although the major Cu^+^ efflux systems have been identified and well characterized, Cu^+^ influx mechanisms are poorly understood. Early characterization of the *E. coli ompB* porin mutant showed a Cu-resistant phenotype, suggesting that Cu^+^ may enter the cells through these outer membrane proteins (Lutkenhaus, 1977). Only recently has the participation of members of the MFS and TonB-dependent transport system in Cu import been proposed (see below). Based on phenotypic analysis, it was previously suggested that some Cu^+^-ATPases might drive metal influx (Odermatt et al., 1993; Koch et al., 2000; Tottey et al., 2001; Lewinson et al., 2009; Hassani et al., 2010). However, direct transport experiments and a better understanding of the Cu^+^-ATPase structure and transport mechanism have provided solid evidence that all P_IB1_-ATPases mediate Cu^+^ efflux (González-Guerrero et al., 2010; Raimunda et al., 2011).

#### Import of copper-chelating molecules

Methane-oxidizing bacteria rely on two MMOs to achieve methanotrophic metabolism (Balasubramanian and Rosenzweig, 2008). The particulate pMMO is located in intracellular membranes and requires Cu for function. Thus, these organisms present an opportunity to identify specific Cu^+^-import mechanisms. Two pathways for Cu entry have been described in the methanotroph *Methylosinus trichosporium* (Balasubramanian et al., 2011). One of these, likely involved in Cu-handling to pMMO, requires the production of the siderophore-like methanobactin (Hakemian et al., 2005). The chalkophore structure is known and the Cu^+^ binding capability extends to Cu removal from minerals (Hakemian et al., 2005; Knapp et al., 2007). Mechanistically, the holo-form is postulated to be taken up via an active TonB dependent transport mechanism (Balasubramanian et al., 2011). A less specific import pathway involves stripped or unchelated Cu^+^ import via an outer membrane porin. However, this mechanism is not required for metallation of pMMO.

#### Copper import catalyzed by secondary carriers

Recently, a novel transporter has been identified based on its requirement for the metallation of *cbb3*-COX. *R. capsulatus* CcoA, a member of the MFS family, has been proposed to function as Cu importer (Ekici et al., 2012b). The MFS family consists of seventy-three sub-families of transporters that catalyze the symport, antiport or uniport of a wide variety of substrates, dissipating chemical or electrochemical gradients (Reddy et al., 2012). Like other MFS, CcoA is predicted to have twelve TM helices divided into two subdomains of six helices each and separated by a large cytoplasmic loop. Similar to the eukaryotic CTR-type Cu^+^-importers, CcoA contains several transmembrane Met rich motifs associated with Cu^+^ binding and transport (Puig et al., 2002; Eisses and Kaplan, 2005; Ekici et al., 2012b). Importantly, mutation of a highly conserved tyrosine in the transmembrane YFLMLIFMT motif of the yeast CTR-type Cu^+^ importer leads to a decrease in Cu^+^ transport (Eisses and Kaplan, 2005). In the CcoA transporter, a similar sequence (Y^230^ALMNLVMT) is also present on the TM7 (Ekici et al., 2012b). Phenotypic characterization of *R. capsulatus* cells lacking a functional CcoA has implicated this transporter in Cu^+^ import pathways, as well as in Cu^+^ acquisition by *cbb3*-COX. Mutation of *R. capsulatus ccoA* leads to a decrease in the total Cu^+^ content of *R. capsulatus* and a decrease in the assembly and stability of the subunits of *cbb3*-COX without inactivating the periplasmic MCO (CutO) (Ekici et al., 2012b). The similar phenotypic characteristics of the *P. aeruginosa* mutant Δ*copA2* (missing the FixI/CopA2-like Cu^+^-ATPase) (González-Guerrero et al., 2010) and the *R. capsulatus* Δ*ccoA* mutant strains, together with their opposed direction of Cu^+^-transport, support a model with COX metallation depending on the efflux of cytosolic Cu^+^ (González-Guerrero et al., 2010; Ekici et al., 2012a).

## Chaperones and chelators

### Cytoplasmic chaperones

Bacterial Cu^+^-chaperones, CopZs, are involved in cytoplasmic Cu^+^ trafficking. Structurally, these proteins present a classic βαββαβ ferredoxin-like folding with an invariant GXXCXXC Cu^+^-binding motif (Banci et al., 2004, 2010a,b; Boal and Rosenzweig, 2009). Extensive studies have shown their interaction with the regulatory N-MBD of Cu^+^-ATPases, exchanging Cu^+^ with K_eq_ ≈ 1 (Argüello et al., 2007; Boal and Rosenzweig, 2009; Banci et al., 2010a,b; Robinson and Winge, 2010). Similarly, they exchange the metal with Cu^+^ sensors (Cobine et al., 1999), and probably can receive the metal from bacterial Cu^+^ importers. These interactions appear mediated by metal-dependent electrostatic interactions (Arnesano et al., 2001, 2002a; Boal and Rosenzweig, 2009; Banci et al., 2010a,b). An example of the importance of electrostatic interactions is the mechanism by which the CopZ loads Cu^+^ substrates into transmembrane transport sites of Cu^+^-ATPases (González-Guerrero and Argüello, 2008; González-Guerrero et al., 2009; Argüello et al., 2011; Padilla-Benavides et al., 2013b). In this case, the electrostatic docking between the negatively charged surface of CopZ with the electropositive platform region of CopA directs the chaperone Cu^+^-binding residues toward the “entrance” of the TM-MBS (Gourdon et al., 2011; Padilla-Benavides et al., 2013b). As the ion moves within the enzyme to the TM-MBS, the chaperone is unable to receive the transported metal back from the enzyme.

A new role for Cu^+^-chaperones has been proposed in *Halobacterium salinarum* (Pang et al., 2013). In this organism, high Cu^+^ induces temporal changes on transcription rates of two Cu^+^-chaperones and one Cu^+^-transporting ATPase through the activation of a Mer-like transcription factor. As observed previously in *P. aeruginosa*, the Cu^+^ responsive metalloregulator is not up-regulated by Cu^+^ (Teitzel et al., 2006; Raimunda et al., 2013). This, plus the fact that pools of small Cu^+^ sequestering molecules like glutathione do not compete with the metalloregulator for Cu^+^ (Changela et al., 2003), implies that a regulatory feedback involving physical interaction and Cu^+^ transfer between the chaperones and the regulator might exist. The study proposed that one CopZ would play this role limiting Cu^+^ access to the metalloregulator. This would allow the repression of the Cu^+^-transporting ATPase transcription, maintaining a hypothetically required intracellular Cu^+^ quota. Accordingly, the cytosolic Cu^+^ increase observed in Cu stressed chaperone mutant cells, points to a dual role of the chaperone participating in the sensing mechanisms (interaction with pools and metalloregulator) and delivering Cu^+^ to the CopA1-like ATPase.

Finally, CupA, a novel membrane-bound Cu^+^ chaperone was identified in *Streptococcus pneumoniae* (Fu et al., 2013). This pathogenic bacterium lacks the “classical” CopZ-type Cu^+^-chaperone. The soluble domain of CupA is able to interact with and deliver Cu^+^ to CopA N-MBD. Consequently, it was suggested to function as a membrane-bound Cu^+^-chaperone contributing to Cu^+^ homeostasis. Surprisingly, CupA adopts a cupredoxin-like folding with a binuclear Cu site accessible and flexible enough to allow Cu^+^ exchange. The finding of a folding more suited for electron transfer functions, instead of the classical ferredoxin-like observed in previously described bacterial Cu^+^-chaperones, is an intriguing variation that opens the possibility of novel functions.

### Periplasmic chaperones

Cu distribution and dynamics in the periplasmic space are complex. The presence of different cuproenzymes such as azurin (Raimunda et al., 2013), CueO (Roberts et al., 2002), Cu/Zn-Sod (Gort et al., 1999), laccases (Claus, 2003), cytochrome *c* oxidases (Richter and Ludwig, 2003), and tyrosinases (Claus and Decker, 2006) among other cuproproteins, exemplifies the vast diversity of periplasmic Cu^+^-binding proteins in different bacterial species (Figure 1). Although the function of these enzymes is fairly understood, the mechanisms underlying their metallation and Cu^+^ traffic in this compartment require further studies. Experimental evidence has shown that both Cu^+^-ATPases and periplasmic chaperones, such as *Thermus thermophilus* PCu(A)C and *R. capsulatus* Sco/SenC, are required for the assembly of functional cytochrome *c* oxidases (Swem et al., 2005; Abriata et al., 2008; González-Guerrero et al., 2010; Lohmeyer et al., 2012). However, the metal trafficking pathways from either the ATPases or the chaperones to the target proteins are not known. A similar novel example of Cu^+^ trafficking within the periplasm is the metallation of *S. enterica* sv. Typhimurium SodCII (Osman et al., 2013). Here, the participation of the periplasmic chaperone CueP appears necessary. In this case, two Cu^+^-ATPases, CopA and GolT, seem able to provide Cu^+^ for metallation of SodCII.

The role of the periplasmic chaperone CusF, associated with the CusCFBA efflux system, in the efflux of periplasmic Cu^+^ has also been described. CusF interacts with and delivers Cu^+^ to CusB, which will further translocate the metal to the extracellular milieu (Mealman et al., 2011). Less is known about the periplasmic Cu^+^-chaperones from the Pco system. Bioinformatics analysis suggests the existence of two periplasmic chaperones: PcoA and PcoC. PcoA is a MCO (Djoko et al., 2008) that may functionally replace CueO in some bacterial systems where CueO is not present (Hernández-Montes et al., 2012) or under microaerobiosis, when this enzyme is not active (Outten et al., 2001; Rensing and Grass, 2003). Double mutation of *cueO* and *cusCFBA* in *E. coli* GR10 cells rendered a hypersensitive response to Cu^+^; complementation of this strain with a plasmid encoding for *pcoA* restored Cu^+^ tolerance of these cells (Lee et al., 2002). No specific function has been proposed for PcoC, though this putative periplasmic protein binds one Cu^+^-equivalent (Lee et al., 2002). Whether or not these chaperones interact with the putative transporting components of the Pco system (PcoB and PcoD) remains to be elucidated. It is evident that within the bacterial periplasm, a vast diversity of periplasmic chaperones and enzymes have fundamental roles in maintaining Cu^+^ homeostasis in coordination with the different efflux and influx systems. Thus, highlighting the necessity of further experimental and metalloproteomics integrative studies.

### Other cytoplasmic chelators and uncharacterized bacterial copper pools

How much Cu^+^ in the surrounding environment can a cell withstand? Gram-negative bacteria, such as *P. aeruginosa* and *E. coli*, replicate at normal rates when grown at high Cu^+^ concentrations (1–2 mM). Such high Cu^+^ tolerance might be explained by the fact that bioavailable Cu^+^ (or Cu^+^ available in the media) might be several orders of magnitude below that range. However, cells could take up chelated-Cu^+^ as a silent piggyback rider while bound to nutrients or, more likely, by specialized Cu^+^ import systems after being stripped off the ligand molecule by specific exchange reactions. Both mechanisms would secure the Cu^+^ quota. However, the second mode of Cu^+^ entry would require the proper storage and sorting once Cu^+^ reaches the cytoplasm. In this regard, there is a lack of information on the constituents and sizes of Cu^+^ pools in the cell. Interestingly, it has been shown that oxidative and nitrosative stress, conditions where Cu plays a central role as part of the stress-tolerance machinery, triggers synthesis of cytosolic metallothioneins (Gold et al., 2008) and other extracellular cuproproteins (Raimunda et al., 2013). Thus, these proteins might have important functions not only in Cu^+^ storage, but also in sensing mechanisms. Recent work in *H. salinarum* suggests an interplay between Cu pools and Cu^+^-chaperones with relevant implications in Cu^+^ homeostasis (Pang et al., 2013).

## Toward integration

Previous sections have described the different elements participating in Cu homeostasis, including not only those that move the ion within and across compartments, but the various cuproproteins that require the metal for function. The requirement to maintain the cell free of Cu^+^ determines that the metal is transferred and delivered to final targets via chelator/protein-protein interactions. The molecular details of some of these events are not well understood and are just started to be uncovered. Similarly, although numerous examples are recorded in the literature, the presence of alternative arrangements of transporters and chelators has not been considered in an integrated fashion. These aspects are discussed in the following sections.

### Achieving selectivity: Cu^+^-ATPases and chaperones

As pointed out, it is accepted that Cu^+^ ions are not free in cellular systems because of the high affinity binding of chaperone, sensing, and transport proteins. It is clear that these “high affinities” are for Cu^+^ binding/release into the aqueous media and they do not represent the molecular affinity for Cu^+^ when the ion is located at the interacting interphase of two partner proteins “exchanging” Cu^+^. Thus, Cu^+^ dependent protein-protein recognition is key for selectivity. An example of this phenomenon is the Cu^+^ transfer from the cytoplasmic chaperones to the TM-MBS of transport ATPases. In this case, the specific electrostatic complementation of the Cu^+^-bound chaperone with ATPase appears to determine the interaction (Padilla-Benavides et al., 2013b). As a consequence, structurally similar MBDs carrying a different electrostatic surface cannot deliver Cu^+^ to the ATPase nor can the apo-form of the chaperone “compete” with its holo-form (González-Guerrero and Argüello, 2008).

This parsimonious model might, however, be challenged in organisms with more than one Cu^+^-ATPase gene (Padilla-Benavides et al., 2013b). Thus, it could be hypothesized that in the genomes of organisms with more than one Cu^+^-ATPase gene might also contain genes for different Cu^+^-chaperones. Additionally, there may be alternative small proteins or other molecules that deliver Cu^+^ for transport through a particular ATPase.

### Achieving selectivity: transporters as targeting mechanisms

While protein-protein interaction is a likely determinant for compartmental Cu^+^ distribution to various target molecules, directing Cu^+^ through distinct transporters also appears to play a central role (Figure 1). An example is again provided by alternative roles described for Cu^+^-ATPases. Bacterial genomes contain at least one gene encoding for a Cu^+^-ATPase, which is essential in conferring Cu^+^ tolerance (Rensing and Grass, 2003; Osman and Cavet, 2008; Argüello et al., 2011). However, the presence of multiple Cu^+^-ATPase coding genes in some genomes suggests their participation in different cellular processes. The idea of redundancy conferring reliability has been considered to understand the presence of multiple Cu^+^-ATPases. However, this increases complexity and energetic cost. In some cases, such as *Salmonella*, phenotypical and functional differences amongst two Cu^+^-ATPases, CopA and GolT, are a matter of debate (Checa et al., 2007; Osman et al., 2010). However, new subgroups of functionally distinct, non-redundant Cu^+^-ATPases have been described (Raimunda et al., 2011).

For instance, the *P. aeruginosa* genome encodes for two non-redundant Cu^+^-ATPases: CopA1 and CopA2 (Figure 1). CopA1 is the classical Cu^+^-ATPase, and its deletion leads to Cu^+^ accumulation and sensitivity for this metal. CopA2 presents slow kinetics of transport and higher affinity for Cu^+^ than that the observed in classic Cu^+^ detoxifying enzymes (González-Guerrero et al., 2010). This slow rate of transport is incompatible with a role in Cu^+^-detoxification but seems adequate to contribute to the assembly of cuproproteins. CopA2/FixI-like ATPases are co-transcribed with COX subunits. As such, it has been demonstrated that in *P. aeruginosa* CopA2 is required for the activity of this cuproprotein (González-Guerrero et al., 2010). One question immediately emerges, beyond these frequently observed functions (Cu^+^ detoxification and COX metallation) should we expect the distinct participation of Cu^+^-ATPases in other processes? Considering the presence of up to five Cu^+^-ATPase genes in certain bacterial genomes, it is tempting to hypothesize that unique/specific roles determined by their kinetic characteristics and structural determinants will emerge for each. As a corollary, if each Cu^+^-ATPase has a distinct function the simultaneous presence of multiple periplasmic chaperones serving different subgroups of cuproproteins is also necessary. In this hypothetical model, the presence of multiple cytoplasmic Cu^+^-chaperones might also be expected.

### Alternative architectures for Cu^+^ efflux systems: copper homeostasis in γ-proteobacteria

Analysis of Cu transport and distribution in various bacteria has shown the frequent presence of polycistronic “transport systems” (See Maintaining the Cu^+^ quota: Cu^+^-sensing and transcriptional regulation of homeostatic systems). Recent bioinformatics analyses of systems involved in periplasmic homeostasis have shown that only 3% of the available γ-proteobacteria genomes present the full set of Cu homeostasis systems previously described (Rensing and Grass, 2003; Osman and Cavet, 2008; Hernández-Montes et al., 2012). That is, the vast majority of the organisms analyzed lack one or more components in one of these systems. While the Cu^+^-ATPases appear universal, the presence of other components seems to cluster in independent groups or clades: CueP, PcoC-YebZ-CutF-CusF-CueO, PcoE-PcoD, PcoA-PcoB, and CusC-CusA-CusB. This suggests that, depending on the presence or absence of some components, bacteria achieve Cu homeostasis through different strategies (Hernández-Montes et al., 2012). Since the selective pressure produced by the host immune system favored and preserved these detoxification mechanisms, it is not surprising that some pathogenic bacteria, such as *Klebsiella pneumonia, Enterobacter cloacae, E. coli, Cronobacter sakazakii*, and *Cronobacter turicensis*, carry the largest amount of proteins required for Cu homeostasis in their genomes (Hernández-Montes et al., 2012). Importantly, the linkage among components seems evolutionarily driven by protein-protein interactions rather than by function. For instance, CusC is distributed independently of CusB and CusA, as it is the most encountered protein after CopA in the clades. Alternatively, CueP was found in organisms also containing CusCFBA, arguing against the hypothesis that the former compensates for the latter in *Salmonella*. This presents challenges to any integrative parsimonious description of Cu homeostasis.

## Future directions

Significant insights into the metal selectivity, regulation, transport mechanism, and interaction with metallochaperones have been gained. The high-resolution crystal structure of the Cu^+^-ATPase, various cytoplasmic and periplasmic chaperones, Cu^+^ sensors, and several components of the Cus system, as well as their biochemical characterization, has contributed to the elucidation of structure-function relationships in these molecules. The interaction between cytosolic Cu^+^ chaperones with the ATPases, as well as the access of Cu^+^ to the TM-MBSs, is relatively well understood. However, little information is available on the interaction of chaperones and sensors, or how the chaperones obtain Cu^+^ from their transmembrane transporters and subsequently deliver it to cuproenzymes. Despite the fact that biochemical studies will soon target these questions, a higher order of integration appears necessary to understand the homeostasis of this micronutrient. While global transcriptomics studies have provided some progress toward this goal, it appears that establishing the total composition of the cuproproteomes is an important requirement. Subsequently, a systems biology analysis, taking into account Cu^+^ pools sizes, their kinetics and thermodynamics, seems to be the next logical step.

### Conflict of interest statement

The authors declare that the research was conducted in the absence of any commercial or financial relationships that could be construed as a potential conflict of interest.
